# Methicillin-Resistant *Staphylococcus capitis* with Reduced Vancomycin Susceptibility Causes Late-Onset Sepsis in Intensive Care Neonates

**DOI:** 10.1371/journal.pone.0031548

**Published:** 2012-02-14

**Authors:** Jean-Philippe Rasigade, Olivia Raulin, Jean-Charles Picaud, Charlotte Tellini, Michele Bes, Jacqueline Grando, Mohamed Ben Saïd, Olivier Claris, Jerome Etienne, Sylvestre Tigaud, Frederic Laurent

**Affiliations:** 1 INSERM U851, National Reference Center for Staphylococci, University of Lyon, Lyon, France; 2 Department of Clinical Microbiology, Northern Hospital Group, Hospices Civils de Lyon, Lyon, France; 3 Department of Neonatology, Northern Hospital Group, Hospices Civils de Lyon, Lyon, France; 4 Department of Hygiene and Epidemiology, Edouard Herriot Hospital Center, Hospices Civils de Lyon, Lyon, France; 5 Department of Neonatology, Eastern Hospital Group, Hospices Civils de Lyon, Lyon, France; The University of Hong Kong, Hong Kong

## Abstract

**Background:**

Coagulase-negative staphylococci, mainly *Staphylococcus epidermidis*, are the most frequent cause of late-onset sepsis (LOS) in the neonatal intensive care unit (NICU) setting. However, recent reports indicate that methicillin-resistant, vancomycin-heteroresistant *Staphylococcus capitis* could emerge as a significant pathogen in the NICU. We investigated the prevalence, clonality and vancomycin susceptibility of *S. capitis* isolated from the blood of NICU infants and compared these data to adult patients.

**Methodology/Principal Findings:**

We conducted a retrospective laboratory-based survey of positive blood cultures in NICU infants ≥3 days of age (n = 527) and in adult ICU patients ≥18 years of age (n = 1473) who were hospitalized from 2004 to 2009 in two hospital centers in Lyon, France. *S. capitis* was the most frequent pathogen in NICU infants, ahead of *S. epidermidis* (39.1% vs. 23.5% of positive blood cultures, respectively). Conversely, *S. capitis* was rarely found in adult ICU patients (1.0%) compared to *S. epidermidis* (15.3%). *S. capitis* bloodstream isolates were more frequently resistant to methicillin when collected from NICU infants than from adult patients (95.6% vs. 53.3%, respectively). Furthermore, we collected and characterized 53 *S. capitis* bloodstream isolates from NICU infants and adult patients from six distant cities. All methicillin-resistant *S. capitis* isolates from NICU infants were clonally related as determined by pulsed-field gel electrophoresis. These isolates harbored a type V-related staphylococcal chromosomal cassette *mec* element, and constantly showed either vancomycin resistance (37.5%) or heteroresistance (62.5%). Conversely, the isolates that were collected outside of the NICU were genetically diverse and displayed much lower rates of vancomycin resistance and heteroresistance (7.7% and 23.1%, respectively).

**Conclusions/Significance:**

A clonal population of methicillin-resistant *S. capitis* strains has spread into several French NICUs. These isolates exhibit reduced susceptibility to vancomycin, which is the most widely used antimicrobial agent in the NICU setting.

## Introduction

Constant improvements in neonatal intensive care have led to better survival rates for very-low-birth-weight infants (VLBW, <1500 g), but the high incidence of nosocomial, late-onset sepsis (LOS, occurring after 3 days of age) is still a leading cause of morbidity and mortality [1], [2]. Coagulase-negative staphylococci (CoNS) are the most frequently encountered pathogens in bloodstream infections in neonatal intensive care units (NICUs) [3], [4]. Several risk factors for such infections have been identified, including low gestational age, low birth weight [5], parenteral nutrition with intravenous lipids, and the presence of a central venous catheter [6], [7]. Although CoNS bloodstream infections are significantly less severe than those caused by other pathogens [4], they lead to prolonged hospitalization and increased antibiotic use [4], [8].

*Staphylococcus epidermidis* has been shown to be the predominant species in CoNS bacteremia in both adult and pediatric patients [9]–[12]; in the NICU setting, it represents 70% of CoNS isolates [12]–[15]. Among non-*epidermidis* CoNS, *Staphylococcus capitis* is rarely, if ever, isolated from bacteremic adult patients [16]. Although this species has been occasionally reported in infective endocarditis cases [17], [18], it is most often considered to be a blood culture contaminant [16]. However, in the NICU setting, recent studies have indicated that methicillin-resistant *S. capitis* could emerge as a significant pathogen, causing LOS in VLBW infants [14], [19]–[22]. The high prevalence of methicillin-resistant CoNS in NICUs usually leads to frequent vancomycin use. Concerns have been raised about the spread of vancomycin-heteroresistant *S. capitis* strains in NICUs and their involvement in persistent bacteremia despite prolonged vancomycin therapy [19], [20], [23].

The unusually high prevalence of methicillin-resistant *S. capitis* bloodstream infections in the NICUs of the University Hospital of Lyon, France, prompted this study. The objectives were: (1) to describe the species distribution of bloodstream CoNS isolates in these NICUs; (2) to investigate the clonality of *S. capitis* that causes LOS in the NICUs of Lyon, and compare it to *S. capitis* strains found in adult patients and in other French NICUs; and (3) to investigate vancomycin resistance and heteroresistance in these strains.

## Methods

### Retrospective prevalence study

We conducted a retrospective laboratory-based survey of blood cultures at two NICUs located in two different hospital centers within the University Hospital of Lyon, France. Both NICUs provide third-level neonatal care, with approximately 70% (Northern Hospital Group) and 55% (Eastern Hospital Group) of infants having VLBW. The inclusion period was from January 1, 2004 to December 31, 2009. The microbiological records of blood cultures drawn from NICU infants after the third day of life (to exclude early-onset sepsis) were obtained from the computerized databases of the clinical microbiology laboratories of each hospital. For comparison purposes, the blood culture results of patients over 18 years of age who were hospitalized in the ICUs of these hospitals during the study period were also reviewed. The first positive blood culture result from each patient was considered for analysis. We also determined the number of patients who had different blood cultures positive with different pathogens. Both participating microbiology laboratories performed species-level identification of the bacterial isolates and antimicrobial susceptibility testing (AST) with the automated BD Phoenix system (Becton Dickinson, Sparks, MD). The AST results were interpreted according to the recommendations of the French Society for Microbiology [24].

Because the study was laboratory-based and no clinical data were obtained, CoNS-positive blood cultures were interpreted to be probable or possible CoNS bacteremia based on the number of positive blood cultures and the patient setting (i.e., NICU infants or adult ICU patients). In both NICU infants and adult ICU patients, probable CoNS bacteremia was defined as ≥2 blood culture sets positive with the same CoNS species within a 3-day period [25]. CoNS-positive blood cultures in adult ICU patients that did not match these criteria were excluded (n = 585). However, because repeated blood cultures are not frequently performed in NICU infants compared to adult patients, single CoNS-positive blood cultures in NICU infants were interpreted as possible CoNS bacteremia and included for analysis. Seventeen CoNS-positive blood cultures were excluded because they were explicitly recorded as false positives in the microbiological record, and thus not identified at the species level. Finally, we excluded blood cultures that were positive with organisms considered to be contaminants other than CoNS, including *Bacillus* spp. (n = 4 NICU infants and 10 ICU patients) and *Micrococcus* spp. (n = 1 NICU infant and 11 ICU patients).

The resistance patterns of *S. capitis* to penicillin, methicillin, gentamicin, erythromycin, clindamycin, pristinamycin, rifampin, fusidic acid and fluoroquinolones were recorded and compared between NICU and adult ICU isolates. Two records were excluded because AST results were incomplete and the corresponding isolates could not be recovered from the strain collection of the microbiology laboratory. The results regarding vancomycin susceptibility were excluded from the analysis because the ≤4 mg/L clinical breakpoint used during the 2004–2009 inclusion period differed from the ≤2 mg/L clinical breakpoint recommended since 2010 by the European Committee on Antimicrobial Susceptibility Testing (EUCAST) [26]. Because the study consisted of a retrospective review of routine microbiological data that were analyzed anonymously, approval by the ethics committee and informed consent were not required.

### *S. capitis* isolates

To investigate the population structure of *S. capitis* at the regional level, *S. capitis* isolates were collected retrospectively among routinely stored clinical strains from participating centers in distant cities spanning the French territory, namely Caen, Limoges, Lyon, Saint-Etienne, Troyes and Versailles. In each laboratory, all bloodstream isolates with the exception of isolates interpreted as contaminants were stored regardless of their resistance profile, according to the French guidelines on bacterial strain storage [27]. *S. capitis* bloodstream isolates from NICU infants and adult/older pediatric patients regardless of their methicillin resistance status were included. The inclusion period was from January 2006 to April 2009 for the isolates collected from the Northern Hospital Group of Lyon, and from January 2008 to April 2009 for the isolates collected from the other participating centers because of the shorter duration of routine storage of clinical strains in these laboratories. Because the strain collection at Lyon consisted of a large number of *S. capitis* NICU isolates (n = 125), selection was restricted to 25 isolates using a random sampling algorithm without replacement by means of the XLStat software version 7.5.2 (Addinsoft SARL, Paris, France). Additionally, an *S. capitis* strain that was involved in a previously reported *S. capitis* outbreak at the NICU of Nantes from 2000 to 2003 was kindly provided by J. Caillon and also included in the study [22]. A total of 53 isolates were investigated, including 40 isolates from NICU infants (Lyon, n = 25; Limoges, n = 7; Saint-Etienne, n = 3; Caen, n = 2; Troyes, n = 2; and Nantes, n = 1) and 13 isolates from adult/older pediatrics patients (Lyon, n = 12; and Versailles, n = 1). Species-level identification of all *S. capitis* isolates included in this study was confirmed by amplification and sequence analysis of the *tuf* gene as previously described [28].

### Pulsed-field gel electrophoresis

All *S. capitis* isolates (n = 53) were characterized by PFGE as previously described [29]. Genomic DNA was digested with *Sma*I restriction endonuclease, which discriminates well between staphylococci, including *S. capitis* [30]. After staining with ethidium bromide, electrophoresis gels were photographed and analyzed with GelCompar software version 4.1 (Applied Math NV, Saint-Martens-Latem, Belgium) using the unweighted pair group method with arithmetic mean (UPGMA). The Dice correlation coefficient was used to determine the similarity percentage between DNA restriction patterns, which were assigned to pulsotypes using >80% similarity with a band position tolerance of 1% as previously described [31]. The genetic diversity of the isolates from NICU infants was compared with the isolates from adult/older pediatric patients using Simpson's index of diversity (SID), which is defined as the percentage probability that two isolates chosen at random are unrelated [32].

### Methicillin resistance and staphylococcal chromosomal cassette *mec* typing

The susceptibility of the 53 selected isolates to oxacillin and cefoxitin was determined by the standard agar diffusion technique as recommended by the French Society for Microbiology [24]. SCC*mec* typing was performed on a subset of 23 methicillin-resistant isolates representative of each pulsotype and geographic origin. Multiplex PCRs were performed to determine the allelic variants of both the chromosomal recombinase *ccr* gene complex cassette and the *mec* locus according to the method established by Kondo et al. [33]. Each combination of *ccr* and *mec* alleles was related to an SCC*mec* type when applicable, following the recommendations of the International Working Group on the Classification of Staphylococcal Cassette Chromosome Elements (IWG-SCC) [34].

### Vancomycin resistance and heteroresistance testing

Vancomycin minimum inhibitory concentrations (MICs) were determined using the E-Test method (AB Biodisk, Solna, Sweden) with a McFarland density 0.5 inoculum on Mueller-Hinton agar as recommended by the manufacturer. The E-Test method was preferred to the CLSI broth microdilution reference method because of its better performance at detecting staphylococci with reduced vancomycin susceptibility [35], [36]. Strains exhibiting a vancomycin MIC>2 mg/L were considered to be resistant according to EUCAST breakpoints [26]. Strains with a vancomycin MIC≤2 mg/L were tested for vancomycin heteroresistance using the brain heart infusion (BHI) screen agar method as previously described [37]. Briefly, an overnight blood agar plate culture was suspended in 0.9% saline and adjusted to McFarland 0.5 turbidity. Four 10-µL droplets of the suspension were dropped using a pipette onto a BHI agar plate (Difco, Becton Dickinson) containing 16 g/L pancreatic digest of casein (Bacto, Becton Dickinson) and 4 µg/mL vancomycin. Plates were incubated at 35°C and the colonies in each droplet were counted after 48 h. A strain was considered to be heteroresistant to vancomycin if ≥1 droplet had ≥2 colonies. The vancomycin-susceptible *S. aureus* strain ATCC 29213 was used as a negative control [37]. The vancomycin-heteroresistant *S. aureus* strain Mu3 and the vancomycin-resistant *S. aureus* strain Mu50 were used as positive controls [38]. The results from each experiment were recorded only when positive and negative controls were confirmed.

### Statistical analysis

The differences in the prevalence of each bacterial species in the positive blood cultures between patient groups and in the antimicrobial resistance rates were analyzed using a two-tailed Fisher's exact test. *P* values were corrected for multiple testing using the Holm-Bonferroni method where appropriate. The differences in vancomycin MICs were analyzed using a non-parametric two-tailed Mann-Whitney *U*-test. *P* values of ≤0.05 were considered to be statistically significant. All statistical tests were performed using the XLStat software version 7.5.2.

## Results

### Methicillin-resistant *S. capitis* is the leading cause of LOS in NICU infants

**Figure 1:**
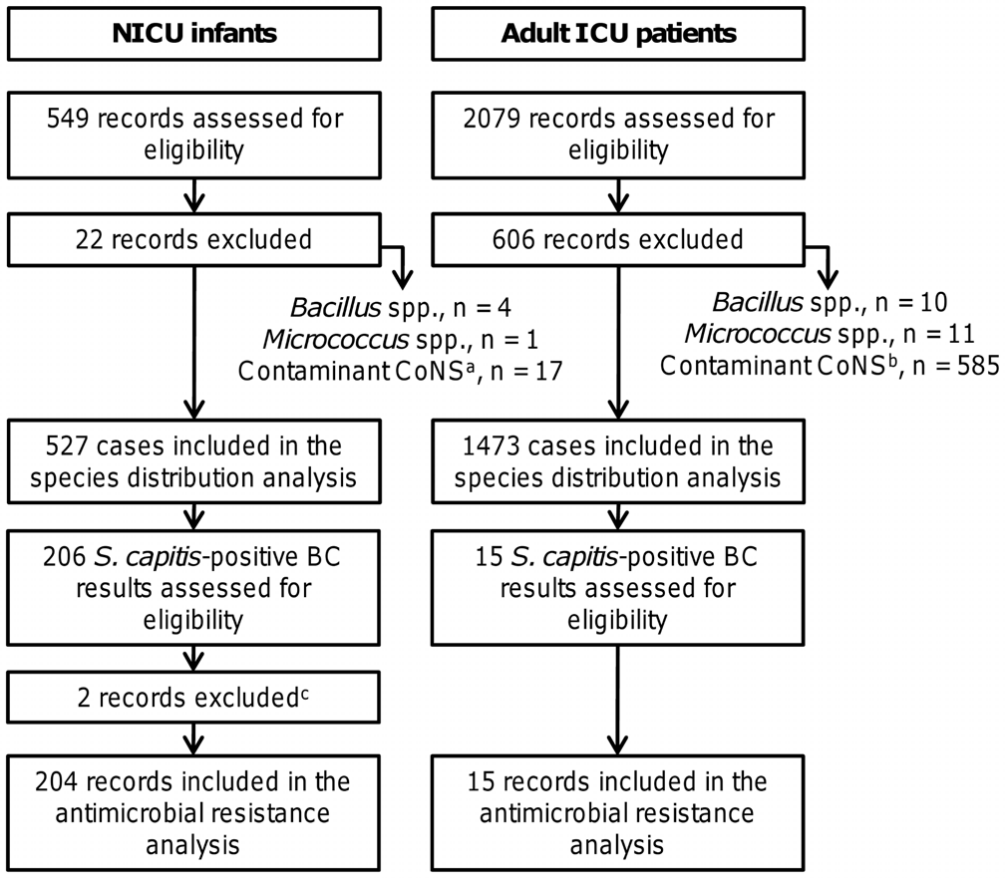
Flow diagram of case selection. (N)ICU, (neonatal) intensive care unit; CoNS, coagulase-negative staphylococci; BC, blood culture. ^a^In NICU infants, a single CoNS-positive blood culture was interpreted as possible bacteremia and included in the analysis. The CoNS-positive results that were excluded were explicitly recorded as a contaminant in the microbiology record. ^b^In adult patients, a single CoNS-positive blood culture was interpreted as a contaminant and excluded from the analysis. ^c^Records were excluded when antimicrobial susceptibility results were not available.

In total, 2,628 patients with positive blood cultures were identified from the laboratory databases, and 2,000 cases were included in the final analysis (Fig. 1). Blood cultures were more likely to grow CoNS when drawn from NICU infants than from adult ICU patients (*P*<0.001, two-tailed Fisher's exact test with Holm-Bonferroni correction; Table 1). *S. capitis* was the most prevalent pathogen that caused bloodstream infections in NICU infants (39.1%) but accounted for only 1.0% of positive blood cultures from adult ICU patients (*P*<0.001). Overall, 54 out of 206 (26.2%) *S. capitis*-positive blood cultures from NICU infants fulfilled the definition for probable CoNS sepsis (≥2 blood culture sets were positive with the same species within a 3-day period), and 152 (73.8%) blood cultures were thus considered to be possible *S. capitis* sepsis. These proportions did not differ significantly from the non-*capitis* CoNS-positive blood cultures (probable sepsis, n = 41/210, 19.5%; possible sepsis, n = 169/210, 80.5%; *P* = 0.13). There was no significant evolution of *S. capitis* prevalence from 2004 to 2009 in either group of patients (data not shown).

**Table 1:** A species distribution comparison of *Staphylococcus* spp. isolates from the positive blood cultures of patients in neonatal intensive care units (NICUs) and adult ICUs, 2004–2009.

	No. (%) of blood cultures (one per patient)			
Blood culture result	NICU infants	Adult ICU patients	*P* value^a^	Odds ratio (95% CI)
Total	527	1473		
*S. aureus*	65 (12.3)	166 (11.3)	0.525	1.11 (0.82–1.50)
Coagulase-negative staphylococci	416 (78.9)	331 (22.5)	<0.001	12.9 (10.1–16.5)
*S. epidermidis*	124 (23.5)	226 (15.3)	<0.001	1.70 (1.33–2.17)
*S. capitis*	206 (39.1)	15 (1.0)	<0.001	62.4 (36.4–106.8)
Other CoNS species	86 (16.3)	90 (6.1)	<0.001	3.00 (2.19–4.10)

a The differences between the groups were tested for statistical significance using a two-tailed Fisher's exact test. *P* values were corrected for multiple testing using the Holm-Bonferroni method.

The overall proportion of patients who had ≥2 blood cultures positive with ≥2 different pathogens was significantly lower in NICU infants than adult ICU patients (n = 177/527, 33.6%, vs. n = 960/1473, 65.2%, respectively; *P*<0.001). Among patients whose first positive blood culture grew *S. capitis*, NICU infants were significantly less likely than adult ICU patients to have subsequent blood cultures positive with another pathogen (n = 49/206, 23.8%, vs. n = 8/15, 53.3%, respectively; *P* = 0.03). Finally, among patients whose first positive blood culture grew a pathogen other than *S. capitis*, NICU infants were significantly more likely than adult ICU patients to have subsequent blood cultures positive with *S. capitis* (n = 42/321, 13.1%, vs. n = 16/1458, 1.1%, respectively; *P*<0.001).

The antimicrobial resistance patterns of *S. capitis* bloodstream isolates from NICU infants (n = 204) and adult ICU patients (n = 15) were compared. The isolates exhibited specific antimicrobial susceptibility patterns depending on the patient setting (Table 2). NICU isolates were more frequently resistant to beta-lactams and gentamicin (*P*<0.001) than adult ICU isolates and more frequently susceptible to erythromycin and fluoroquinolones (*P*<0.05). Among the NICU isolates, the methicillin-resistant isolates were more frequently resistant to gentamicin (*P*<0.001), and more frequently susceptible to fusidic acid than were methicillin-susceptible isolates (*P*<0.05; Table 3).

**Table 2:** A retrospective comparison of the antimicrobial resistance profiles of *Staphylococcus capitis* bloodstream isolates from patients in neonatal intensive care units (NICUs) and adult ICUs, 2004–2009.

	No. (%) of resistant isolates			
Antimicrobial agent	NICU isolates(n = 204)	Adult ICU isolates(n = 15)	*P* value^a^	Odds ratio (95% CI)
Penicillin	202 (99.0)	10 (66.7)	<0.001	50.5 (8.70–293.1)
Methicillin	195 (95.6)	8 (53.3)	<0.001	19.0 (5.63–63.9)
Gentamicin	194 (95.1)	3 (20.0)	<0.001	77.6 (18.8–319.7)
Erythromycin	29 (14.2)	7 (46.7)	0.022	0.19 (0.06–0.56)
Clindamycin	21 (10.3)	4 (26.7)	0.227	0.32 (0.09–1.08)
Pristinamycin	13 (6.4)	1 (6.7)	1.000	0.95 (0.12–7.82)
Rifampin	91 (44.6)	3 (20.0)	0.205	3.22 (0.88–11.8)
Fusidic acid	11 (5.4)	4 (26.7)	0.050	0.16 (0.04–0.57)
Fluoroquinolones	10 (4.9)	7 (46.7)	<0.001	0.06 (0.02–0.20)

a The differences between the groups were tested for statistical significance using a two-tailed Fisher's exact test. *P* values were corrected for multiple testing using the Holm-Bonferroni method.

**Table 3:** A retrospective comparison of the antimicrobial resistance profiles of methicillin-resistant and methicillin-susceptible *Staphylococcus capitis* bloodstream isolates from patients in neonatal intensive care units, 2004–2009.

	No. (%) of resistant isolates			
Antimicrobial agent	Methicillin-resistant isolates (n = 195)	Methicillin-susceptible isolates (n = 9)	*P* value^a^	Odds ratio (95% CI)
Penicillin	195 (100.0)	7 (77.8)	<0.001	NC^b^
Gentamicin	192 (98.5)	2 (22.2)	<0.001	224.0 (32.1–1561.3)
Erythromycin	27 (13.8)	2 (22.2)	1.000	0.56 (0.11–2.85)
Clindamycin	21 (10.8)	0 (0.0)	1.000	NC
Pristinamycin	13 (6.7)	0 (0.0)	1.000	NC
Rifampin	89 (45.6)	2 (22.2)	0.966	2.94 (0.60–14.5)
Fusidic acid	8 (4.1)	3 (33.3)	0.049	0.09 (0.02–0.41)
Fluoroquinolones	10 (5.1)	0 (0.0)	1.000	NC

a The differences between the groups were tested for statistical significance using a two-tailed Fisher's exact test. *P* values were corrected for multiple testing using the Holm-Bonferroni method.

b NC, not calculable.

### *S. capitis* isolates from distant NICUs are clonal and harbor a type V-related SCC*mec* element

**Figure 2:**
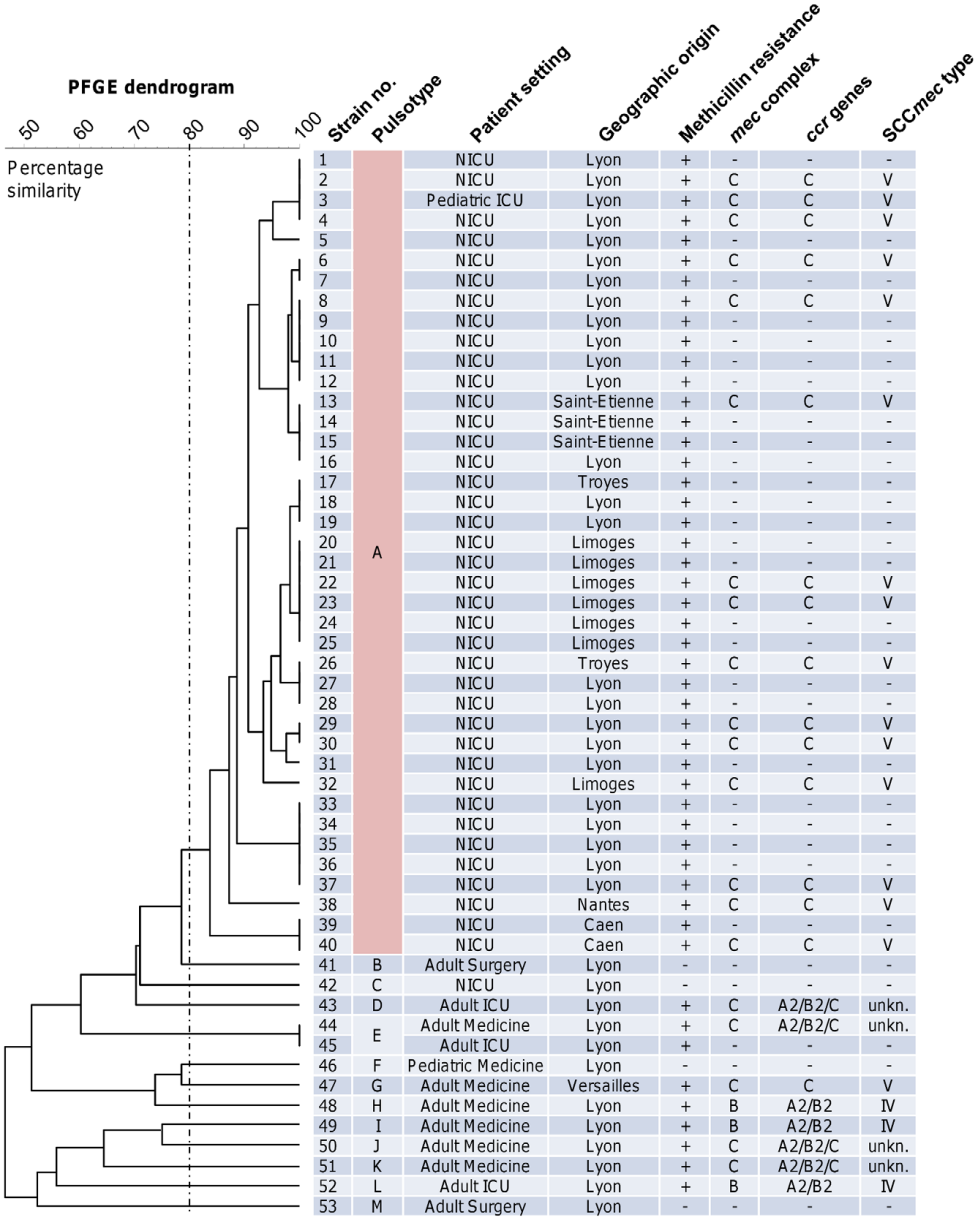
*Staphylococcus capitis* isolates from distant neonatal intensive care units (NICUs) are clonal. Pulsed-field gel electrophoresis (PFGE) was applied to 53 bloodstream isolates of *S. capitis* that were collected from NICU infants and adult patients from cities spanning the French territory. The PFGE dendrogram was generated using the GelCompar software version 4.1. The isolates were assigned to pulsotypes using >80% similarity (vertical dashed line). Staphylococcal chromosomal cassette *mec* (SCC*mec*) typing was applied to 23 methicillin-resistant isolates representative of each pulsotype and geographic origin. All methicillin-resistant isolates from the different NICUs belonged to the same pulsotype and shared a type V-related SCC*mec* element, whereas methicillin-susceptible and/or non-NICU isolates were genetically diverse. *ccr*, chromosomal cassette recombinase; unkn., unknown (a combination of the *mec* complex and *ccr* genes has not been assigned to an SCC*mec* type so far).

Of 53 selected isolates, 49 (92.5%) were resistant to methicillin, including 39 NICU isolates (97.5%) and 10 non-NICU isolates (76.9%). PFGE identified 13 different pulsotypes, designated as NRCS-A to -M (Fig. 2). Except for a single methicillin-susceptible isolate, all NICU isolates that were collected from 7 different NICUs in 6 different cities belonged to the same pulsotype (NRCS-A). In contrast, the 14 remaining isolates, including 1 methicillin-susceptible NICU isolate and 13 isolates from adult or older pediatric patients, belonged to 13 different pulsotypes (NRCS-A to -M). The only NRCS-A isolate that was not from a NICU infant was collected from a 4-year-old patient who was hospitalized in the pediatric ICU of Lyon. Simpson's index of diversity was 4.9% (95% CI, 0.0–14.3%) for the NICU isolates and 91.1% (95% CI, 88.0–94.2%) for the adult/older pediatric isolates.

SCC*mec* typing was applied to a subset of 23 methicillin-resistant *S. capitis* isolates representative of each pulsotype and geographic origin, and all NRCS-A isolates were found to share the *mec* complex C and *ccr* C genes, whose combination matches the features of SCC*mec* type V (Fig. 2) [34]. The same *mec* complex/*ccr* gene combination was only found in one pulsotype NRCS-G isolate from an adult patient from Versailles. The 7 remaining methicillin-resistant isolates from adult patients belonged to pulsotypes NRCS-D, -E, and -H to -L and were found to harbor either the *mec* complex B/*ccr* A2B2 (matching SCC*mec* IV) or the *mec* complex C/*ccr* A2B2C (a combination that has not yet been assigned to an SCC*mec* type to our knowledge).

### NRCS-A isolates have reduced vancomycin susceptibility

**Figure 3:**
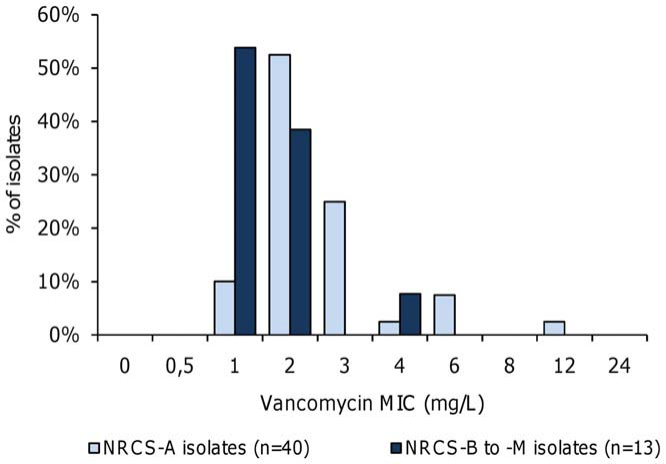
Vancomycin MICs are higher in *Staphylococcus capitis* pulsotype NRCS-A than in other pulsotypes. Vancomycin MICs were determined by the E-Test method. The mean vancomycin MIC was significantly higher in pulsotype NRCS-A isolates (2.8 mg/L) than in pulsotypes NRCS-B to –M isolates (1.7 mg/L), as illustrated by the shift toward the higher values of the MIC distribution curve (*P*<0.01, two-tailed Mann-Whitney *U*-test).

The mean vancomycin MIC of NRCS-A isolates was significantly higher (2.8 mg/L, range 1.5–12 mg/L) than the NRCS-B to -M isolates (1.7 mg/L, range 1–4 mg/L; *P*<0.01, two-tailed Mann-Whitney *U*-test) (Fig. 3). Regarding vancomycin susceptibility, as interpreted using the EUCAST 2010 recommendations [39], the proportion of vancomycin-resistant isolates was 4.9-fold higher in pulsotype NRCS-A than in the other pulsotypes (37.5% vs. 7.7%, respectively; *P* = 0.08, Fisher's exact test). Moreover, the proportion of vancomycin-heteroresistant isolates was 2.7-fold higher in NRCS-A than in the other pulsotypes (62.5% vs. 23.1%, respectively; *P*<0.05). All NRCS-A isolates with a vancomycin MIC in the susceptible range were heteroresistant. Finally, the proportion of isolates exhibiting either vancomycin resistance or heteroresistance was significantly higher in pulsotype NRCS-A than in the other pulsotypes (100.0% vs. 30.8%, respectively; *P*<0.001).

## Discussion

Several major findings emerged from this retrospective study of 2,000 positive blood cultures from NICU infants and adult ICU patients from 2004 to 2009 and from the molecular characterization of a panel of *S. capitis* clinical isolates. First, methicillin-resistant *S. capitis* was the most prevalent pathogen that caused LOS in the NICUs of Lyon, France, whereas this species was rarely isolated from the blood of adult ICU patients. Second, all methicillin-resistant *S. capitis* bloodstream isolates collected from several NICUs spanning the French territory belonged to the same pulsotype, which was designated as pulsotype NRCS-A, whereas the methicillin-susceptible isolates and the isolates from adult and older pediatric patients exhibited high genetic diversity. Third, pulsotype NRCS-A isolates constantly showed either vancomycin resistance (37.5%) or heteroresistance (62.5%), whereas these rates were 4.9- and 2.7-fold, respectively, lower in the isolates collected outside of the NICU. Collectively, these findings indicate that a clonal population of methicillin-resistant *S. capitis* exhibiting reduced susceptibility to vancomycin has spread into several NICUs in France.

*S. capitis* has been sporadically incriminated in NICU outbreaks [22], but the prevalence of this pathogen as a cause of LOS in French NICUs had not been investigated prior to this study. Several surveillance programs, such as RAISIN and NEOCAT [40], [41], have focused on healthcare-associated bloodstream infections in French ICUs and NICUs. However, *S. capitis* prevalence could not be inferred from the results of these studies because CoNS prevalence was not reported at the species level.

Interestingly, previous reports of *S. capitis* in the NICU setting have shown strains of this species to be clustered in a single pulsotype [13], [42]. It is unknown whether the spread of *S. capitis* NRCS-A is limited to France or if these strains are present in the NICUs of other countries. Molecular typing and a comparison of the isolates from international sources are warranted to address this question.

The reasons underlying the success of *S. capitis* pulsotype NRCS-A in the NICU environment are not completely understood. NICU isolates were found to be specifically resistant to the antimicrobial agents used in these wards, namely beta-lactams, aminosides and vancomycin [43], but not fluoroquinolones (Table 2). This adapted resistance profile likely results from a slow and progressive expansion of this clone in NICUs rather than from a rapid epidemic spread. Heteroresistance to vancomycin has been proposed as an intrinsic feature of the *S. capitis* species [23]. An increased tendency towards vancomycin resistance in pulsotype NRCS-A isolates may have been a decisive feature that led to their selection under high vancomycin pressure in the NICUs.

Whether *S. capitis* LOS in NICU infants is associated with higher morbidity or treatment failure compared to other CoNS pathogens is unknown. The present study was specifically designed to address the prevalence and molecular characteristics of *S. capitis* in the NICUs; therefore, we did not investigate the clinical course of *S. capitis* bacteremia. Our findings suggest the need for a prospective cohort study to determine whether specific risk factors are associated with LOS due to *S. capitis* and to what extent elevated vancomycin MICs in this pathogen correlate with an impaired response to glycopeptide therapy.

Our study has limitations in addition to those inherent to its retrospective design that need to be addressed. First, we used laboratory-based definitions of CoNS bacteremia in NICU infants, which determined that one CoNS-positive blood culture was considered to be bacteremic in this population. It is likely that a non-negligible proportion of CoNS-positive blood cultures included in the analysis were false-positive, thus introducing a bias towards an overestimation of CoNS prevalence as a cause of bacteremia. Nonetheless, it is unlikely that the large difference in *S. capitis* prevalence between NICU and adult ICU patients can be explained by this inclusion bias. Second, although we have shown that the *S. capitis* pulsotype NRCS-A is present in the NICUs of several French cities, our prevalence study was limited to the NICUs of a single town; therefore, it does not necessarily reflect the regional epidemiology of *S. capitis*. Third, the over-representation of isolates from Lyon in our collection introduces a potential underestimation of genetic diversity. However, because all of the NICU isolates collected outside of Lyon belonged to pulsotype NRCS-A, we can reasonably conclude that this sampling bias does not account for the observed clonality of *S. capitis* in the NICUs. Finally, one cannot exclude the possibility that this clonality resulted from a lack of discriminatory power of *Sma*I PFGE regarding the *S. capitis* species, but the high diversity of the PFGE banding patterns in the isolates from adult patients does not support this hypothesis. Furthermore, additional typing of a subset of NRCS-A isolates from different cities using repetitive sequence-based PCR [44] yielded indistinguishable banding patterns (data not shown) that confirmed the close genetic relationship between these isolates.

To conclude, the *S. capitis* pulsotype NRCS-A has adapted remarkably to the NICU setting; it has overtaken *S. epidermidis* as the leading pathogen that causes LOS in the NICUs of Lyon while remaining virtually absent from adult patients. Microbiologists and physicians in NICUs with high *S. capitis* prevalence should be aware of the potential reduced vancomycin susceptibility of this pathogen.
